# Potential of High-Intensity Focused Ultrasound in Enamel Remineralization

**DOI:** 10.1177/00220345251323869

**Published:** 2025-03-19

**Authors:** B. Shrestha, S.M. Rajan, M. Saunders, A. Fawzy

**Affiliations:** 1 UWA Dental School, The University of Western Australia, WA, Australia; 2 Centre for Microscopy, Characterisation and Analysis, The University of Western Australia, WA, Australia

**Keywords:** ultrasonic energy, minimal invasive dentistry, enamel white spot lesions, enamel repair, cariogenic biofilms, biomaterials

## Abstract

Remineralization is an essential interventional strategy for intercepting enamel white spot lesions (WSLs). Given the limitations of both natural and/or fluoride-mediated repair processes, there is a need to develop novel strategies for repairing enamel WSLs via a minimally invasive approach while restoring the unique ultrastructural integrity and functional properties. Inspired by the unique capability of high-intensity focused ultrasound (HIFU) in facilitating the crystallization process, we propose a novel strategy of employing HIFU for in vitro repair of WSLs through synergizing the crystallization process required for hydroxyapatite (HAP) formation from its precursor (calcium phosphate ion clusters; CPICs). Following CPIC formulation and characterization including the resultant amorphous calcium phosphate (ACP), the effect of HIFU on the ACP-to-HAP transition on the amorphous substrate was investigated using transmission electron microscopy and high-resolution transmission electron microscopy, selected area electron diffraction, and X-ray diffraction (XRD). The results showed profound amorphous-to-crystalline phase transition, within 5- to 30-min HIFU exposure, whereas the long axis of the resultant HAP corresponded with the (002) plane, and a lattice spacing of 0.34 nm indicated a preferred *c*-axis growth direction consistent with the orientation of natural enamel crystallites. For enamel repair, artificial WSLs were created on enamel specimens and then subjected to CPICs, followed by HIFU exposure for 2.5, 5, or 10 min. Scanning electron and atomic force microscopies revealed the decreased surface roughness and the gradual obliteration in the WSL porous structure with continuous linear coaxial arrangement of HAP crystallites filling the prismatic/interprismatic gaps closely resembling sound enamel specifically with 5-min HIFU exposure. Enamel WSL ultrastructural repair was further confirmed from XRD and Raman spectral analyses with the associated regaining of mineral density and nanomechanical properties as reflected from micro–computed tomography (CT) and nanoindentation results, respectively. Micro-CT further validated the subsurface remineralization of WSLs with HIFU exposure. Within the same exposure parameters, HIFU exhibited a potent antibiofilm effect against *Streptococcus mutans*. This study introduced a new approach for remineralizing enamel WSLs through the potent synergy between HIFU and CPICs.

## Introduction

In minimally invasive dentistry, remineralization is an important strategy for managing early carious lesions. Although saliva facilitates natural remineralization, its effectiveness after progression to white spot lesion (WSL) has been questioned (Van der Veen et al. 2007). Consequently, fluoride-mediated remineralization was introduced as a preventive approach (Ten Cate 2004). Nevertheless, there is an increasing risk of fluoride-associated toxicity with fluoride intake from multiple sources (Martínez-Mier 2012; Zohoori and Maguire 2018). Moreover, studies have reported that fluoride-mediated remineralization compromises the mechanical properties of the repaired enamel, because it does not have the ability to form ordered mineral crystals (Fan et al. 2009). Second, the formation of fluorapatite hinders mineral ion diffusion into deeper regions of the lesion (Bubnowicz and França 2022), resulting in incomplete enamel remineralization. Given the limitations of both natural and fluoride-mediated repair processes, there is a need to develop novel strategies for repairing tooth enamel with WSLs via a minimally invasive approach while restoring the unique ultrastructural integrity and functional properties.

Researchers are actively exploring innovative interventions for the remineralization of early enamel demineralized lesions such as hydroxyapatite (HAP) formation from amorphous calcium phosphate (ACP) nanoparticles as precursor assembly, protein/peptide–driven mineralization, and hydrogel-induced remineralization (Li et al. 2011; Ruan et al. 2014; Mukherjee et al. 2018). However, these approaches are performed under strict conditions including high temperature and pressure, low pH, technical complexities, and/or high cost, which could limit their translation to wide-scale clinical practice.

It is worth nothing that HAP crystallites in natural enamel originate from ACP precursors, specifically composed of Posner’s clusters (Ca_9_[PO_4_]_6_), which are ion clusters with a domain size of approximately 0.95 nm (Posner and Betts 1975; Müller et al. 2022). Therefore, calcium phosphate ion clusters (CPICs) have been proposed as a mineralization frontier for enamel remineralization (Shao et al. 2019). Despite showing epitaxial growth, CPIC-mediated enamel repair may not be clinically viable because the crystallization method requires approximately 48 h of incubation under controlled conditions. In addition, this approach was investigated only for repairing phosphoric acid demineralized lesions as a study model, which did not simulate the actual early enamel carious lesions.

**Figure 1.**
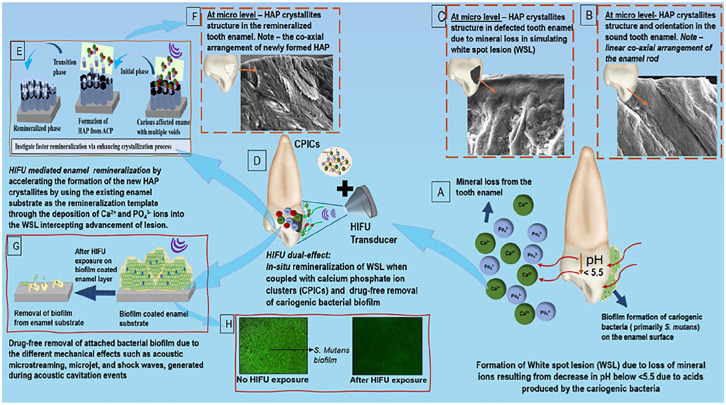
Schematic demonstration of the proposed concept of the potential of high-intensity focused ultrasound (HIFU) in synergizing the remineralization process of artificially simulated enamel carious lesion (white spot lesion; WSL) coupled with calcium phosphate ionic clusters (CPICs), along with its associated drug-free antibiofilm effect. (**A**) Mineral loss from the tooth enamel due to a reduction in pH associated with an acidic oral environment induced by cariogenic bacterial biofilms. (**B**) Scanning electron microscopy image showing the structure and orientation of hydroxyapatite (HAP) crystallites in sound tooth enamel. (**C**) Continuous exposure to an acidic environment resulting in mineral loss from HAP, affecting its enamel prismatic structure and leading to the formation of WSLs. (**D**) Schematic presentation summarizing the proposed dual action of HIFU in synergizing the remineralization of enamel WSL and eradicating the associated cariogenic biofilm. (**E**) The acoustic cavitation event generated by HIFU results in both chemical and physical effects (including the formation of highly reactive radical species, shockwaves, and microjets) enabling the accelerated formation of HAP crystallites in the presence of CPICs for repairing enamel WSLs (**F**). Schematic presentation (**G**) and confocal laser scanning microscopy images (**H**) showing the drug-free eradication of cariogenic biofilms attached to the enamel surface through HIFU’s mechanical effect.

High-intensity focused ultrasound (HIFU) has gained wide attention in medicine and dentistry as a promising therapeutic modality, due to its unique interaction with biological tissues and the drug-free antimicrobial properties (Fawzy et al. 2019; Chen et al. 2022; Rajan et al. 2023). HIFU is an intense focused ultrasonic energy capable for generating acoustic cavitation, resulting in both physical effects (such as shock waves and high-velocity microjets) and/or chemical effects (such as the formation of free radical species; Sander et al. 2014).These properties have been reported to accelerate crystallization processes while controlling the size and shape of the synthesized nanoparticles (Sander et al. 2014; Yusof and Ashokkumar 2015). In addition, sonochemical methods have shown to be promising approaches for the synthesizing HAP nanoparticles due to their ability to enhance nucleation process, decrease crystallization time, and improve purity (Bang and Suslick 2010). Inspired by the unique capability of HIFU in facilitating the crystallization process (Shrestha et al. 2024) with the associated drug-free antibiofilm potential (Rajan et al. 2023), herein, we propose a novel strategy based on the concept of using HIFU to synergize the in vitro repair of enamel WSLs following the application of CPICs (Fig. 1). Therefore, the first aim is to assess the effect of HIFU exposure on the transformation of noncrystalline ACP to crystalline HAP nanoparticles. The second aim is to investigate the synergistic effect of HIFU and CPICs on repairing enamel specimens bearing artificial WSLs. The final aim is to evaluate the antibiofilm effect of HIFU against cariogenic bacterial biofilms attached to the enamel surface.

## Materials and Methods

### Nanoparticle Synthesis and Characterization

CPICs were synthesized according to Shao et al. (2019). ACP nanoparticles were obtained by air drying the CPIC–ethanol solution for 15 min. The resultant nanoparticles were characterized by transmission electron microscopy (TEM)–energy dispersive spectroscopy (EDS; JEOL F200 CF-HR). The chemical composition was confirmed using Fourier transform infrared attenuated total reflectance spectroscopy (FTIR-ATR; IR-Spirit) at a spectrum ranging between 4,000 and 400 cm^−1^. The biocompatibility of the synthesized CPICs was investigated using human oral fibroblast (Aati et al. 2022).

### HIFU Experimental Setup

**Figure 2.**
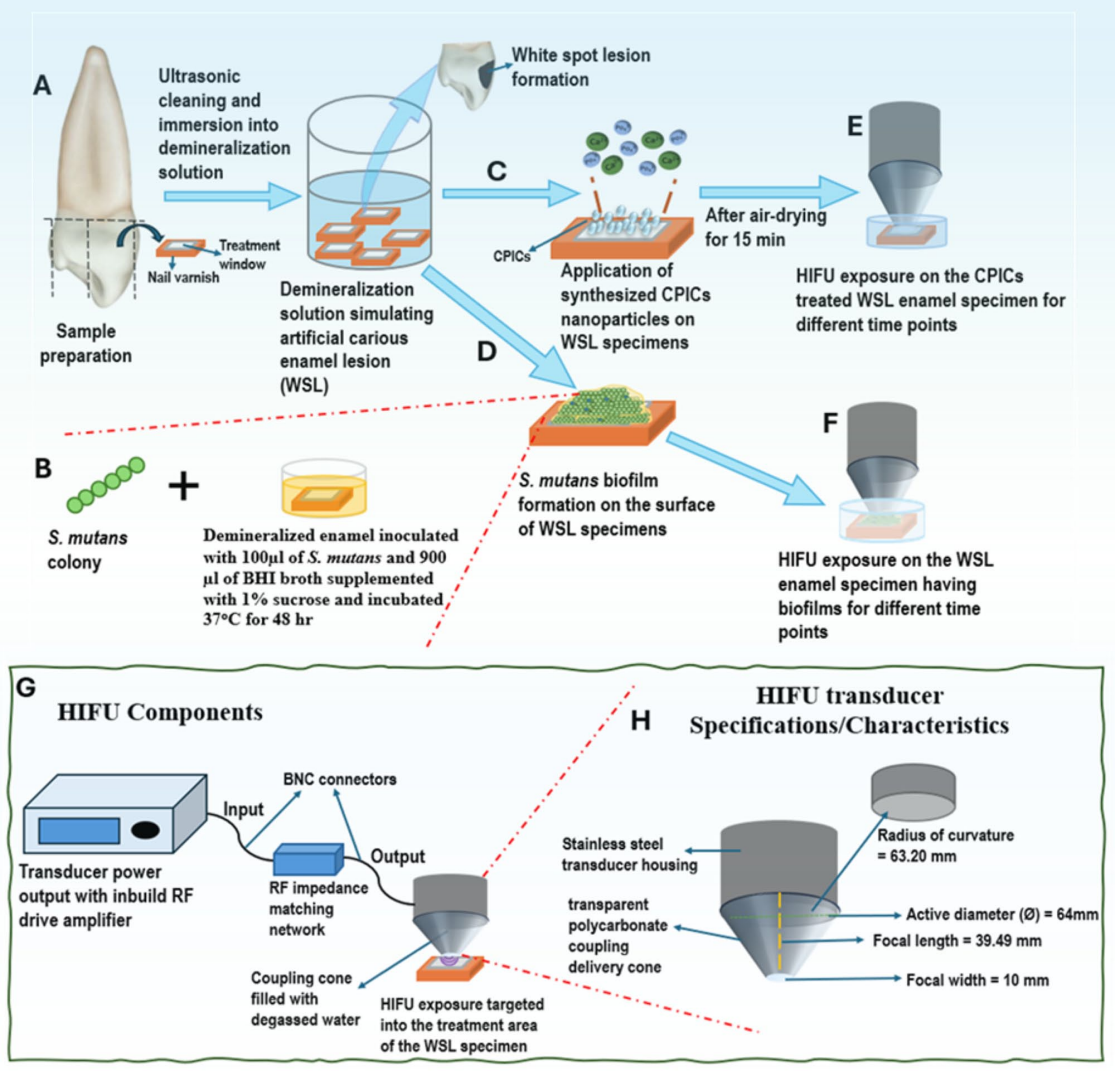
Schematic diagram representing the experimental setup for investigating the synergizing effect of high-intensity focused ultrasound (HIFU) and calcium phosphate ion clusters (CPICs) on enamel remineralization along with characterizing the antibiofilm effect of HIFU on *Streptococcus mutans*. (**A**) Illustration of the methodology for creating an artificially simulated white spot lesion (WSL). (**C**) Application of synthesized CPICs on the artificially created enamel WSL, followed by subjecting the specimens to HIFU with 250 KHz operated at 30 W (continuous mode) to evaluate the synergistic effect of HIFU and CPICs for remineralizing WSL enamel (**E**). (**B**, **D**) Cultivation of *S. mutans* biofilm on the WSL enamel, followed by HIFU exposure for different time points (**F**). (**G**) HIFU components and attachments including a bowl-shaped piezoelectric ceramic transducer with a resonance frequency of 250 KHz, attached to a transparent polycarbonate coupling cone for acoustic delivery, along with HIFU transducer specifications (**H**).

The HIFU setup consists of a bowl-shaped piezoelectric ceramic transducer (H-115; Sonic Concepts) with a resonance frequency of 250 KHz operated at 30 W (continuous mode) attached to a polycarbonate coupling cone (C-101, Sonic Concepts; Fig. 2). The coupling cone (which contained degassed water) was used to synchronize and deliver the focused waves to the specimens.

### Crystallization Potential of HIFU

The effect of HIFU on the ACP-to-HAP transition on amorphous glass substrates was investigated. Distilled water was added to glass vials containing ACP nanoparticles generated from CPICs. Each vial was positioned at the focal point and exposed to HIFU for different durations (2.5 to 30 min). The formed HAP was characterized using an X-ray diffractometer (Malvern Panalytical) across the 2θ range. HAP nanoparticles were analyzed using TEM-EDS, selected area electron diffraction (SAED), and high-resolution (HR) TEM (JEOL F200 CF-HR).

### Synergistic Effect of HIFU and CPICs on Enamel WSL Repair

Human premolars (*N* = 140) were used following ethics approval (Re-2019/RA/4/20/5863). Enamel specimens were prepared from the labial surfaces (4 × 3 × 3 mm) using a low-speed diamond saw (IsoMet) and polished with ascending grit silicon–carbide discs under water coolant, then ultrasonicated for 5 min in distilled water. Artificial WSLs were created (He et al. 2022) and assigned to 5 experimental groups: HIFU exposure without CPIC nanoparticles (HIFU/-NP), CPIC nanoparticles without HIFU exposure (NP/-HIFU), or CPIC nanoparticles followed by HIFU exposure for 2.5 min (NP/HIFU [2.5 min]), 5 min (NP/HIFU [5 min]), or 10 min (NP/HIFU[(10 min]). CPICs carried on ethanol solution (1 mL; 2 mg/mL) were applied to fully cover the WSL and air dried for 15 min and then exposed to HIFU for 2.5, 5, or 10 min.

Enamel surface morphology was viewed using scanning electron microscopy (SEM; Verios XHR/SEM, Thermo-Fisher Scientific). Atomic force microscopy (AFM) scanning (Cypher VRS, Oxford Instruments) was performed in tapping mode at 25 × 25 µm, and surface roughness (S_a_) was calculated. X-ray diffraction (XRD) peaks of the enamel specimens having WSLs were first recorded over the 2θ range. Enamel specimens treated with CPICs before and after HIFU exposure were recoded to investigate any crystallographic changes. Mineral composition analyses were performed by Raman microscopy (WITec alpha 300RA). The Lorentz fitting function was performed to extract the area under the curves (1,070 cm^−1^ and 960 cm^−1^) using Project Five software (version-5.1, WITec).

To assess the mineral density and lesion depth, enamel specimens were scanned using micro-computed tomography (micro-CT; Nikon XT H225 ST CT). To calculate the mineral density, the Hounsfield unit (HU) phantom was calibrated using the same tube (filled with water; water phantom). The HU values for air and water were assigned as −1,000 and 0, respectively, to calibrate the HU scale in CTAn software (version 1.14.4.1, Bruker micro-CT). Thereafter, the mineral density was estimated from the projection data set, in which the regions of interest (ROIs) were selected from 3 different sections of the WSL. The lesion depth was further measured from the same ROI slices. Nanohardness and reduced elastic modulus were characterized using DualScope SPM (DME), equipped with a Berkovich diamond indenter (~20 nm).

### HIFU Antibiofilm Effect

The antibiofilm effect against *Streptococcus mutans* was evaluated using confocal laser scanning microscopy (CLSM; Nikon, USA), metabolic activity (MTT), and colony-forming unit (CFU) assays.

### Statistical Analyses

Data normal distribution was investigated by Shapiro–Wilk test and presented as mean ± standard deviation. Analysis of variance followed by post hoc Tukey’s test was performed at *P* < 0.05 significance using SPSS (version 23.0, IBM-Armonk).

More details are reported in the Appendix.

## Results

### Characterization CPICs and ACP Nanoparticles

**Figure 3.**
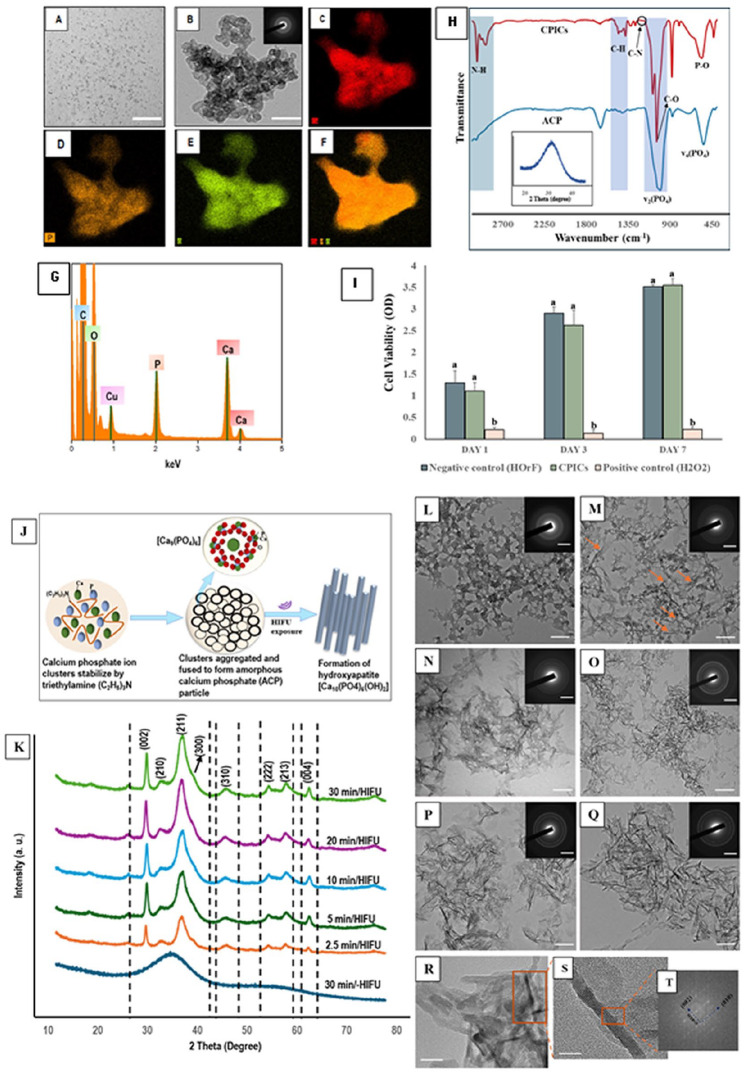
Transmission electron microscopy (TEM) images showing the morphology of the synthesized (**A**) calcium phosphate ion cluster (CPIC) nanoparticles and (**B**) amorphous calcium phosphate (ACP) resulting from the fusion of CPICs (scale bars 200 nm). Inset in (B): selected area electron diffraction (SAED) of the amorphous ACP. (**C–G**) TEM–energy dispersive spectroscopy mapping and elemental analysis of ACP nanoparticles confirming the presence of calcium, phosphorous, and oxygen, respectively. (**H**) The acquired Fourier transform infrared spectra of synthesized CPICs (red spectrum) and ACP nanoparticles (blue spectrum). Inset in (H): X-ray diffraction of ACP reconfirming the amorphous phase. (**I**) Acid phosphatase assay of oral fibroblasts against formulated CPIC nanoparticles at different time points (data are presented as mean and SD; dissimilar letters indicate statistical difference). (**J**) Schematic demonstration of mineral phase transformation leading to hydroxyapatite (HAP) formation facilitated by high-intensity focused ultrasound (HIFU) exposure. (**K**) X-ray diffraction patterns of the HIFU-exposed ACP specimens showing the major diffraction peaks observed with increasing exposure time identified as the (002), (211), (300), (310), (222), (213), and (004) planes confirming the formation of crystalline HAP. (**L–Q**) Corresponding TEM images: (L) no structural changes were observed with the amorphous ACP nanoparticles without HIFU exposure. (M) Following 2.5 min of HIFU exposure, chainlike aggregates begin to partially transform into a needlelike structure. (N–Q) After 5 to 30 min of HIFU exposure, more well-defined needlelike particles became more visible. The respective TEM-SAED (insets) showed an increase in the intensity of the diffraction rings with an increase in HIFU exposure time confirming the transformation of the amorphous precursor to the crystalline phase. (**R, S**) High-resolution TEM (HR-TEM) images and (**T**) the associated fast Fourier transform (FFT) of the formed HAP following 30 min of HIFU exposure showing that the long axis of the nanorod corresponds with the (002) plane, with lattice spacing of 0.34 nm. Scale bar 200 nm for TEM images (L–Q), 5 1/nm for TEM-SAED, and 50 and 20 nm for HR-TEM (R, S).

The TEM investigation showed that CPICs were formed of round particles with an average diameter of 1 to 1.2 nm, consistent with Shao et al. (2019) (Fig. 3A). Upon air drying, these particles were assembled and aggregated to form spherical-shaped ACP (Fig. 3B–F). This was corroborated by FTIR findings (Fig. 3H), wherein the prominent peaks at ~567 and ~1,055 cm^−1^ assigned to O-P-O bending vibration and P-O stretching, respectively (Jin et al. 2021) reflected the conversion from CPICs to ACP. The proliferation rate of human oral fibroblast (HOrF) cells in the presence of CPICs showed no difference compared with untreated HOrF cells, demonstrating their acceptable biocompatibility (Fig. 3I).

### Crystallization Potential of HIFU

The XRD (Fig. 3K) diffraction peaks recorded with or without HIFU exposure were identified as the (002), (211), (300), (222), (213), and (004) planes of HAP by comparing them with a standard HAP reference (COD–96-901-0053), indicating the ACP-to-HAP phase transition. The XRD results were consistent with the TEM investigation (Fig. 3L–Q), wherein most of the amorphous precursors had transformed into crystalline needlelike particles within 5 min of HIFU exposure. Furthermore, the long axis of the resultant HAP particles shown by HR-TEM and the associated fast Fourier transform (FFT) corresponds with the (002) plane, with a lattice spacing of 0.34 nm (Fig. 3T) indicating a preferred *c*-axis growth direction of the synthesized HAP (Di Chen et al. 2007; Jevtic et al. 2008).

### The Synergistic Effect of HIFU and CPICs on Enamel Repair

#### Ultrastructural and crystallographic changes

**Figure 4.**
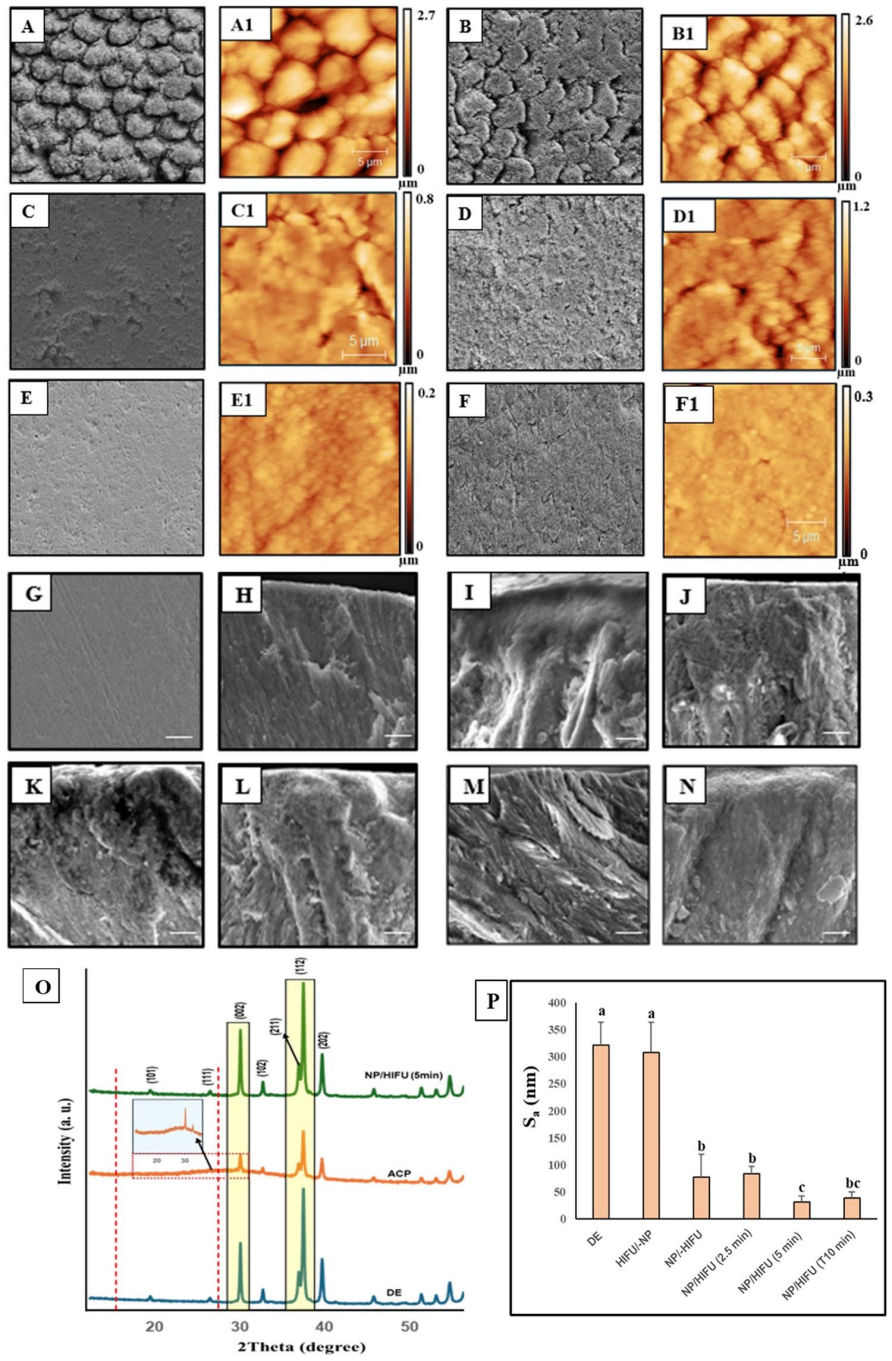
(**A–G**) Scanning electron microscopy (SEM) and (A1–F1) atomic force microscopy (AFM) images of the enamel specimens. (A, A1) Enamel with untreated white spot lesion (WSL; DE) displayed a highly porous surface and interprismatic gaps. (B, B1) Enamel WSL exposed to high-intensity focused ultrasound (HIFU) alone for 10 min demonstrated close morphological similarities to untreated WSL. (C, C1) The application of calcium phosphate ion clusters (CPICs) alone resulted in a smoother surface texture and formation of an amorphous calcium phosphate (ACP) coating layer masking the underlying prismatic and interprismatic structure. Enamel treated with CPICs/HIFU for 2.5 (D, D1), 5 (E, E1), and 10 min (F, F1) exposure time, respectively, revealed a gradual obliteration of the typical prismatic–interprismatic structure. (G) Sound enamel. (**H–N**) The corresponding cross-sectional SEM images of the enamel specimens. (H) Sound enamel showing a well-defined longitudinally oriented prismatic structure with closely packed rodlike hydroxyapatite (HAP) crystallites extending to the outermost surface layer. (I) Untreated WSL displayed a structureless, porous, disorganized surface layer with no defined orientation. More or less similar morphological features to the untreated WSL (I) were found following HIFU exposure alone (J), CPIC application alone (K), and CPIC/HIFU exposure for 2.5 min (L). A well-defined longitudinally oriented prismatic structure having a rodlike morphological appearance was observed with HIFU exposure for 5 min (M) and 10 min (N) following CPIC application. Specifically, CPICs followed by HIFU exposure for 5-min treatment (M) demonstrated a continuous organized linear coaxial arrangement of closely packed HAP crystallites. (**O**) X-ray diffraction (XRD) spectral analyses of enamel specimens. The blue spectrum represents untreated WSL (DE). Specimens treated with CPICs alone (orange spectrum) showed the formation of an amorphous ACP layer reflected from the broad hump in the XRD pattern at the 15 to 30.15° 2θ position (inset). However, following subsequent HIFU exposure for 5 min (green spectrum), the reappearance of a sharp peak within the same 2θ position inferred the conversion of amorphous ACP to crystalline HAP. (**P**) Mean roughness value (S_a_) showing the gradual decrease in surface roughness with the increase in HIFU exposure time (error bars represent SD; dissimilar letters indicate statistically differences). SEM scale bars: 10 µm.

The SEM and AFM images of enamel specimens are presented in Figure 4. The enamel WSL showed a highly porous surface and interprismatic gaps resulting from loss of HAP crystallites due to mineral ion depletion. This pattern remained unchanged, including surface roughness, following HIFU exposure (10 min) without CPIC application (HIFU/-NP). Conversely, the application of CPICs without HIFU exposure resulted in a smoother surface due to ACP masking the effect of the underlying demineralized porous surface pattern, as reflected from the XRD diffraction peak (Fig. 4O). Following the combined CIPC/HIFU treatment, a reduction in surface microporosity, interprismatic gaps, and surface roughness (S_a_) was clearly visible with the increase in exposure time to 5 and 10 min. The restoration of the enamel ultrastructure was apparent with 5-min HIFU exposure following CPIC application and was comparable with sound enamel (Fig. 4E, G, H, M). XRD confirmed this finding by revealing a distinct, sharp intense diffraction peak at a 2θ° of 15 to 30° (Fig. 4O, green spectrum), in comparison with the broad low-intensity peak (Fig. 4O, orange spectrum) recorded with the NP/-HIFU group. This indicates the transformation of ACP (amorphous phase)-to-HAP (crystalline phase) in the enamel specimens treated with CPICs followed by HIFU for 5 min.

#### Mineral composition, density, and nanomechanical properties

**Figure 5.**
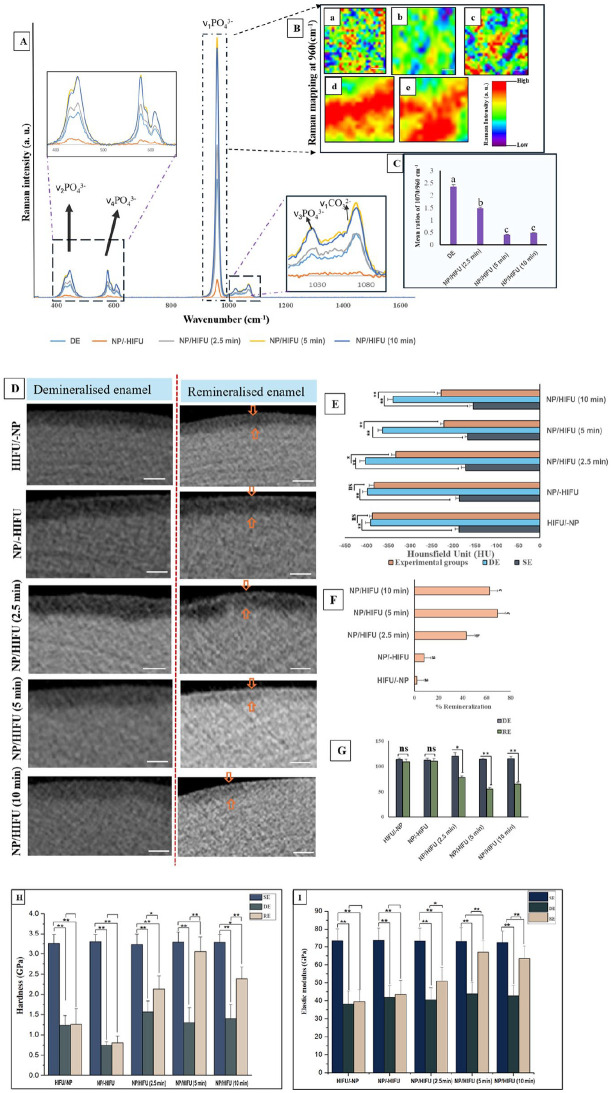
(**A**) Raman spectral analysis showing the intensity increase of the characteristic phosphate peaks (*v_1_* PO_4_^3−^, *v_2_* PO_4_^3−^, *v_4_* PO_4_^3−^) in the experimental groups treated with calcium phosphate ion cluster nanoparticles (NP) followed with high-intensity focused ultrasound (HIFU) for 2.5, 5, and 10 min compared with untreated white spot lesion (WSL; DE), indicating an increase in hydroxyapatite (HAP) content. (**B**) Raman mapping at 960 cm^−1^: (a) WSL showing a decrease in 960 cm^−1^ peak intensity distribution, indicating a reduction in HAP content; (b) NP/-HIFU treated group; and (c–e) the experimental groups (NP/ HIFU [2.5 min], NP/HIFU [5 min], NP/HIFU [10 min]), respectively. The highest HAP density distribution as represented by the red color was shown by the NP/HIFU (5 min) and (10 min) groups. (**C**) Bar chart demonstrating the ratio of the integrated area under the bands of 1,070 cm^−1^ and 960 cm^−1^ showing a significant decrease with the increase in HIFU exposure time depicting the decrease in the carbonate content. (**D**) Representative cross-sectional 2-dimensional micro-computed tomography images of enamel specimens before and after remineralization (scale bars: 300 µm). The orange arrows indicate the difference in the gray-scale intensity. (**E**) Bar chart showing the variation of mineral density represented by (Hounsfield unit) of each treated group in relevance to their respective sound enamel (SE) and WSL (DE). (**F**) Bar chart showing the percentage of the remineralization rate between the different groups. The NP/HIFU (5-min) group exhibited the highest recovery rate (69%) compared with all other experimental groups. (**G**) Bar chart showing the variation in the lesion depth after remineralization interventions. Bar charts showing the variations in (**H**) nanohardness and (**I**) reduced elastic modulus. Experimental groups (NP/HIFU [2.5 min], NP/HIFU [5 min], NP/HIFU [10 min]) showed a significant increase in nanohardness and reduced elastic modulus as compared with the respective WSL control. *Significant difference; ns, no difference. Graphs show mean values and SD; dissimilar letters represent significant difference.

The intensities of the Raman characteristic peaks (960, 587, and 430 cm^−1^) associated with the PO_4_ group in HAP (Carden and Morris 2000) were higher for all CPIC/HIFU groups compared with the WSL control (Fig. 5A), as corroborated by Raman mapping (Fig. 5B). Notably, the Raman spectra of the NP/-HIFU exhibited considerably lower intensities than those of the WSL control, with only a low-intensity peak at 960 cm^−1^ detected (Fig. 5A), consistent with the XRD findings (Fig. 4O). Furthermore, the carbonate concentration, as determined by the 170/969 cm^−1^ ratio, was significantly lower (*P* < 0.05) in the experimental groups (NP/HIFU [2.5, 5, 10 min]) compared with the control (WSL; Fig. 5C).

Micro-CT images showing cross-sectional slices of each group along with the mineral density and lesion depth are depicted in Figure 5D–G. The gray-scale intensities for the HIFU/-NP and NP/-HIFU groups closely matched those of the WSL control (Fig. 5D). The experimental groups, NP/HIFU (5 and 10 min), displayed a more homogenous distribution of increased gray-scale intensity within the demineralized area (Fig. 5D). This was further reflected by the mineral density (Fig. 5E) and lesion depth (Fig. 5G) values, indicating a significant increase in HU and a decrease in the lesion depth with the NP/HIFU (5 and 10 min) groups compared with their respective WSL counterparts, with a remineralization rate of 69% and 62%, respectively (Fig. 5F). Consistent with the micro-CT results, both NP/HIFU (5 and 10 min) groups showed a significant increase in the nanohardness and reduced elastic modulus compared with the WSL control counterparts (*P* < 0.05; Fig. 5H, I).

## Discussion

The crystallization potential of HIFU on the formation of HAP from its ACP precursor was validated through various characterization techniques (Fig. 3). The ACP specimens subjected to HIFU showed an increase in the distinctive sharp diffraction peaks and distinct continuous electron diffraction rings with increasing exposure time, as revealed from XRD (Fig. 3K) and TEM-SAED (Fig. 3M-Q: insets), indicating a profound amorphous-to-crystalline phase transition. This could be further reflected from their morphological characterization (Fig. 3N–Q), confirming the accelerated phase transition, which is likely attributable to the reduced induction time linked to the acoustic cavitation events. Shock waves and microjets generated by acoustic cavitation collapse at the ACP–water interface coupled with induced localized heating effects could explain the accelerated crystallization process (Bang and Suslick 2010). Moreover, HIFU induces the formation of free radicals in water, required for the conversion of ACP to HAP (Kim and Suslick 2018). Furthermore, the *d_hkl_* values calculated from the FFT pattern from HR-TEM correspond to the interplanar spacing of (002) planes for the hexagonal structure (Fig. 3T), indicating a preferred *c*-axis growth direction (Di Chen et al. 2007; Jevtic et al. 2008).

Driven by our previous investigation showing HIFU’s capacity to expedite the formation of crystalline HAP with a similar crystallographic orientation as tooth enamel from ACP when applied to phosphoric acid demineralized enamel (Shrestha et al. 2024), we investigated whether HIFU, when combined with CPICs, exhibits a similar synergistic effect on the remineralization of enamel WSL as a more clinically relevant study model (Figs. 4, 5). Enamel specimens treated with CPICs followed by HIFU for 5 min exhibited ultrastructural features closely similar to sound enamel (Fig. 4). This could be explained by the deposition of Ca^2+^ and PO_4_^3−^ ions and the subsequent formation of HAP crystallites to rebuild the defected enamel structure (Fig. 1E). It is important to note that this observation is not due to the masking effect of the formed ACP layer, as demonstrated by the NP/-HIFU group (Fig. 4C). Instead, it is due to an actual transition from an amorphous to a crystalline phase (Fig. 4O and L).

The CPIC/HIFU synergistic effect on enamel WSL remineralization can be further validated by the significant increase in nanohardness and reduced elastic modulus (Fig. 5H, I), which are explicitly related to the increase in the mineral content (Akkus et al. 2017) and the close alignment of the regenerated HAP crystallites with those of sound enamel (Yilmaz et al. 2015). The increased Raman intensity at 960 cm^−1^ recorded for the NP/HIFU groups indicates a higher HAP content (Fig. 5A). This could confirm that the treated enamel WSLs were remineralized because the ν_1_ band at 960 cm^−1^, which is the most prominent signal in the Raman spectrum of enamel, serves as an indicator of the mineral content (Mohanty et al. 2013). The Raman mapping further revealed that these enhanced ν_1_ bands at 960 cm^−1^ were distributed across a large portion of the repaired enamel surface (Fig. 5B).

Micro-CT analyses were further conducted to evaluate the efficacy of the CPICs/HIFU approach in remineralizing the subsurface zone of the WSL (Fig. 5D–G). This is crucial because the actual enamel caries manifest as subsurface demineralization, owing to the presence of a calcified superficial layer, and often act as a barrier, constraining WSL remineralization to its full depth. The significant increase in the mineral density shown by the NP/HIFU (5 and 10 min) groups reflects that remineralization was not limited to the outer surface layer of the WSL. It also supports the assumption that Ca^2+^ and PO_4_^3−^ from the amorphous nanoparticles infiltrated the subsurface area and transformed into HAP following HIFU exposure. This process ultimately filled the subsurface voids, leading to a reduction in lesion depth and an increase in the mineral density values (Fig. 5E, G). This is in contrast to fluoride-mediated remineralization, wherein the remineralization is limited to the surface layer of the WSL (Bubnowicz and França 2022), leading to surface blocking and leaving behind a porous lesion body inaccessible to remineralizing agents, limiting full lesion consolidation that results in compromised mechanical properties (Fan et al. 2009; Xu et al. 2022). However, due to fluoride’s effect in increasing acid resistance (Sadowsky et al. 1983), further investigations might be necessary to assess whether exposure to fluoride, as a subsequent application step, is recommended for imparting higher acid resistance to repaired enamel.

The ACP-to-HAP transition is dependent on the substrate material on which CPICs was applied followed by HIFU exposure. Therefore, the HIFU exposure time required to optimize such transition was different between enamel (Figs. 4, 5) and glass substrates (Fig. 3) due to the differences in their composition, crystallinity, and ultrastructure. In addition, the flaws and gaps in demineralized enamel serve as a functional template for nucleation sites for Ca^2+^ and PO_4_^3^ ions (Iafisco et al. 2018), facilitating the attachment of ACP. Subsequently, HIFU exposure accelerates the crystal growth of amorphous materials through chemical and/or physical effects such as shock waves, mechanical/shear force, and free radicals generated during acoustic cavitation events (Bang and Suslick 2010; Yusof and Ashokkumar 2015; Kim and Suslick 2018). For instance, the shock wave generated during acoustic cavitation has been shown to reduce the induction time and thereby significantly increase the robustness of the crystallization process, thus promoting crystal growth (Fang et al. 2021). In addition, the microjets created during bubble collapse push the particles against each other, thereby making the nanoparticles oriented parallel and laterally connected within the field of ultrasound streaming (Jevtic et al. 2008).

However, it should be noted that although HIFU exposure for 2.5 min was able to improve the mechanical properties of repaired enamel compared with the WSL counterpart (Fig. 5H, I), it remained significantly lower than the SE control. In addition, with CPIC/HIFU exposure for 10 min, the ultrastructure of the remineralized enamel was not distinctive as compared with the NP/HIFU (5 min) group and SE (Fig. 4N). This was further evident by the decrease in the mineral recovery percentage and relative Raman intensity observed (Fig. 5). This emphasizes the importance of identifying and optimizing the most appropriate HIFU exposure time within the context of all other variables related to substrate materials, acoustic transfer media, lesion study model, and remineralizing agent employed. Therefore, the optimization between those correlated experimental variables is a continuous ongoing process.

The antibiofilm capability of the HIFU is exposure time dependent, as confirmed by CLSM, MTT, and CFU investigations (Appendix Fig. 1). The effect of HIFU against *S. mutans* biofilms is attributed to the combined action of acoustic microstreaming, microjet, and shock waves generated with acoustic cavitation events (Vyas et al. 2019). Furthermore, the drug-free antimicrobial effect associated with HIFU could lower the likelihood of developing resistance, as the bacteria has less time to adapt to stresses from the constant mechanical wave (Vollmer et al. 1998).

It is important to acknowledge that maintaining the long-term integrity of repaired enamel is challenging due to ongoing demineralization/remineralization episodes, which are affected by various factors (Featherstone 2008). Therefore, it is anticipated that CPIC/HIFU-mediated remineralization will need to be complemented with regular oral hygiene measures. As with any treatment intervention, the recurrence of any lesion is influenced by several clinical variables. Therefore, treatment for WSL recurrence and/or developing new lesions that might require reintervention is individually varied and should be considered as part of the patient’s comprehensive treatment plan, including the maintenance phase.

HIFU is nonionization affordable technology that received several Food and Drug Administration approvals for several medical interventions (Focused Ultrasound Foundation 2024). Following concept proofing and identifying the unique HIFU exposure specifications, the customized fabrication of miniaturized HIFU transducers with the associated delivery systems capable of delivering HIFU waves to different locations on the tooth surface and exposing several teeth at the same time is reasonably feasible based on current technological progress in focused ultrasound implementation for medical interventions requiring precise interventions (Prat et al. 2002; Salgaonkar and Diederich 2015). Mounting miniaturized transducers to handheld devices, such as intraoral scanners, with a set of exchangeable tips having different angulations is currently under consideration. Furthermore, array transducers are currently used for applications that require multiple HIFU exposures on several lesions simultaneously (Ramalli et al. 2022). This promotes the fabrication of devices that are similar to sectional impression trays having a set of mounted microarray transducers that facilitate the delivery of HIFU not only on different locations on the tooth but on several teeth simultaneously.

## Author Contribution

B. Shrestha, M. Saunders, contributed to conception, design, data acquisition, analysis, and interpretation, drafted and critically revised the manuscript; S.M. Rajan, contributed to design, data acquisition and interpretation, drafted and critically revised the manuscript; A. Fawzy, contributed to conception, design, data analysis and interpretation, drafted and critically revised the manuscript. All authors gave final approval and agree to be accountable for all aspects of the work.

## Supplemental Material

sj-docx-1-jdr-10.1177_00220345251323869 – Supplemental material for Potential of High-Intensity Focused Ultrasound in Enamel Remineralization Supplemental material, sj-docx-1-jdr-10.1177_00220345251323869 for Potential of High-Intensity Focused Ultrasound in Enamel Remineralization by B. Shrestha, S.M. Rajan, M. Saunders and A. Fawzy in Journal of Dental Research
