# Supplementary material for: Potential of High-Intensity Focused Ultrasound in Enamel Remineralization
Source: J Dent Res. 2025 Mar 19;104(9):983–92. doi: 10.1177/00220345251323869 (PMC12209549; doi:10.1177/00220345251323869)
Supplement: sj-docx-1-jdr-10.1177_00220345251323869 – Supplemental material for Potential of High-Intensity Focused Ultrasound in Enamel Remineralization [file sj-docx-1-jdr-10.1177_00220345251323869.docx]

Supplementary information

**Potential of High-Intensity Focused Ultrasound in Enamel Remineralization**

Barsha Shrestha ^1^, Sheetal Maria Rajan ^1^, Martin Saunders ^2^, Amr Fawzy ^1*^

**Authors’ affiliations**

^1^ UWA Dental School, The University of Western Australia, WA 6009, Australia

^2^ Centre for Microscopy, Characterisation and Analysis, The University of Western Australia, WA 6009, Australia

***Corresponding Author:** Amr Fawzy

UWA Dental School, The University of Western Australia,

17 Monash Avenue, Nedlands WA 6009, Australia

Email: [amr.fawzy@uwa.edu.au](mailto:amr.fawzy@uwa.edu.au)

Tel: +61894572434

**Materials and Methods**

**Synthesis and characterization of the nanoparticles**

The calcium phosphate ionic clusters (CPICs) were synthesized according to previously described methods (Shao et al., 2019). Briefly, solution A was prepared by dissolving 37.5 µl phosphoric acid (H_3_PO_4_; 85% in water solution; sigma-Aldrich, Australia) in 10 ml of ethanol (C_2_H_5_OH; 99.7%; Sigma-Aldrich, Australia). The formed solution was added slowly with slight agitation into solution B consisting of 100 mg of calcium chloride dihydrate (CaCl_2_.2H_2_O; 99.0%; Sigma-Aldrich, Australia) and 1.9 ml triethyl amine [(C_2_H_5_)_3_N; 99.5%; Sigma-Aldrich, Australia) dissolved in 40 ml of C_2_H_5_OH. After the mixture of both the solutions, the final solution appeared as a white cloud-like agitated solution with few minutes. The formed solution was then centrifuged at 30,200 relative centrifuge force (RCF) for 30 min, to obtain gel-like CPIC nanoparticles. Next, ACP nanoparticles were obtained by air-drying the CPIC-ethanol solution for 15 min at room temperature on a glass substrate.

***Morphological and elemental analysis of the synthesized nanoparticles***

The morphology and elemental analysis of the resultant nanoparticles were characterized by transmission electron microscopy (TEM; JEOL F200 CF-HR, Tokyo, Japan) and energy-dispersive X-ray spectroscopy (EDX). The microscope was operated at an acceleration voltage of 200 kV. The nanoparticles were suspended in absolute ethanol and a drop of the suspension was pipetted onto a carbon-coated copper grid (200 mesh) and air-dried before imaging. The images were obtained with a Gatan OneView camera running Gatan Microscopy Suite^®^ (GMS 3, Gatan).

***Spectral analysis of the synthesized nanoparticles***

The chemical composition of the resultant nanoparticles was confirmed using Fourier-transform infrared (FTIR) spectroscopy coupled with attenuated total reflection (ATR). For the analysis, each type of the nanoparticle (i.e., CPICs and ACP) was placed onto a diamond crystal, and a spectrum was recorded with 40 scans at 4 cm^-1^ resolution with spectrum range between 4000 to 400 cm^-1^.

***Cytotoxicity evaluation of the synthesized CPICs nanoparticles***

The biocompatibility of the synthesized CPICs nanoparticles was investigated as previously described protocol (Aati et al. 2022b), with slight modifications using passage 5^th^ human oral fibroblast cells (HOrF; ScienCell Research Laboratories, USA). The cytotoxicity of the synthesized CPICs against HOrF cells was measured utilizing the acid phosphatase (APH) assay, which measures cell viability by quantifying the amount of cytosolic acid phosphatase activity (Aati et al. 2022a). Briefly, HOrF cells were cultured in fibroblast medium supplemented with 2% fetal bovine serum (FBS), 1% antibiotics (penicillin/streptomycin) and 1% fibroblast growth supplement. The culture was incubated at 37 º C with 5 % CO_2_ until 95 % confluence of the cells was achieved. The synthesized CPICs were sterilized under ultraviolet light and then suspended in a fibroblast base medium (FBM; ScienCell Research Laboratories, USA). The HOrF cells were seeded into a 96 -well plate with a 4 x 10^6^ cells/well density and incubated for 24 h. Later, the culture medium was replaced with 200 µl FBM containing CPICs for different time points (1, 3, and 7 days). After each time point, the cells were rinsed with PBS, followed by the addition of 150 µl of APH buffer and incubated for 1.5 h at 37 º C with 5 % CO_2._ Subsequently, 20 µl of 1 M NaOH was added and cell viability was measured at an absorbance of 405 nm by a microplate reader.

**The HIFU experimental set-up**

A single-element, bowl-shaped piezoelectric ceramic transducer (H-115; Sonic Concepts, Bothell, USA), having a resonance frequency of 250 KHz, attached to a transparent polycarbonate coupling cone for acoustic waves delivery (C-101, Sonic Concepts, Bothell, USA) was used in this study (Fig. 2).

An overview of the HIFU set-up (including the technical specifications and dimensions) and schematic presentation of the various experimental configurations done in this study are shown in Figure 2. The transducer was driven by a wave generator connected to an acoustic amplifier (TPO; Sonic Concepts, Bothell, USA) using a custom-made electrical impedance-matching network (50Ω). The customized coupling cone (filled with degassed water as an acoustic transfer media) was used to synchronize and deliver the focused acoustic waves to the specimens at the transducer’s unique focal distance (39.49 mm) and focal width (10 mm) matched to the top-end of the coupling cone. Degassed water was used to make the speed of sound constant over the experimental operation (Fawzy et al. 2019).

Throughout all experimental phases, of this study, HIFU exposure parameters (30 W of continuous exposure mode) were selected based on our pilot investigations to maximize the desired synergizing effect on HAP formation, within clinically feasible intervention times (2.5, 5, and 10 min for enamel specimens), while maintaining the associated temperature rise within the clinically acceptable range. A multichannel thermocouple was placed at the top-end of the collimating coupling cone (where the specimen will be placed) to measure the temperate change occurring during the HIFU exposure for 2.5, 5- or 10-min exposure time. The baseline temperature was 24^0^C, whereas the average temperatures after HIFU operation were 31.19±3.15(2.5 min), 33.35±2.15 (5 min) and 35.22±3.07 (at 10 min HIFU exposure).

**Crystallization potential of HIFU**

***Effect of HIFU on HAP formation***

This experiment was done to study the effect of HIFU exposure on HAP formation from its precursors (CPICs) which were synthesized according to a previously described method (Shao et al. 2019). Distilled water was added to glass vials containing ACP nanoparticles generated from their precursor, CPICs. Each vial was positioned on the top-end of the coupling cone to ensure the correct focal length and exposed to HIFU operated in continuous mode at 30 W for different times points (2.5 min, 5 min, 10 min, 20 min, and 30 min). To serve as control, the nanoparticles were incubated for 30 min at 37°C corresponding to the longest HIFU exposure time used in this study.

***Characterization of the formed HAP***

The phase and composition of the formed HAP nanoparticles, if any, were confirmed by X-ray diffraction (XRD) an Aeris benchtop X-ray diffractometer (Malvern Panalytical, UK) with Co Kα radiation (λ= 1.78 A°) at 40 kV and 15 mA. The data were recorded across a 2θ range of 3 – 80° with a scanning rate of 0.38 sec/step. Furthermore, the morphology, crystallinity, and orientation of the HAP nanoparticles were analyzed using transmission electron microscope (TEM) coupled with energy-dispersive X-ray spectroscopy (EDS), selected area electron diffraction (SAED), and high-resolution TEM (HR-TEM) (JEOL F200 CF-HR, Tokyo, Japan), equipped with a Gatan OneView camera. Samples for TEM imaging was prepared by drop-casting suspensions of particles in ethanol onto carbon-coated copper TEM grids.

**The synergistic effect of HIFU and CPICs on enamel WSL repair**

***Teeth selection and Specimens’ preparation***

Human premolars extracted due to indicated dental reasons were used in this study following patient’s consent and ethical approval from The University of Western Australia Human Research Ethics Committee (Re 2019/RA/4/20/5863). The included premolars were cleaned from any calculus, debris and soft tissues then rinsed in distilled water in ultrasonic bath for 10 min. Teeth were disinfected for 24 h in 0.5% chloramine-T and kept in 0.1% thymol solution and refrigerated at 4 °C until use. The selected teeth were inspected using magnifying lens to ensure that their labial enamel surfaces were free from caries, cracks, or other defects. The included premolars were cleaned from any calculus, debris and soft tissues then rinsed in distilled water in ultrasonic bath for 10 min. Thereafter, sample size calculation was performed utilizing G*Power software (ver. 3.1.9.7; Heinrich-Hein-Universität Düsseldorf, Düsseldorf, Germany); with an effect size of f = 0.4, α err prob = 0.05, and power = 0.95, resulted in total minimum teeth sample size of 140 for the study. Enamel specimens were prepared from the labial surfaces (4 × 3 × 3 mm^3^) of the premolars using low-speed diamond saw (IsoMet, Buehler, Düsseldorf, Germany) under water-coolant. The enamel blocks were then polished with 600-, 800-, 1000-, 1500-, 2000-, and 4000- grit silicon carbide discs, under water coolant, followed by ultrasonication for 5 min twice in distilled water. A two layer of acid-resistant nail varnish was then applied on the polished enamel blocks to outline areas of 4 X 3 mm as treatment windows, followed by storing in 0.1% thymol solution at 4 °C until further experiments.

***White spot lesion formation (WSL) and study groups***

The early enamel carious lesion (White spot lesion; WSL) was created on the prepared enamel specimens as previously described (He et al. 2022). The enamel specimens were immersed in 5.5 ml of demineralization solution (50 mM CH_3_COOH, 2.2 mM Ca (NO_3_)_2_, 2.2 mM KH_2_PO_4_, and 3 mM NaN_3,_ pH of 4.5) and were incubated for 7 days in a shaker at 50 rpm at 37 °C. Thereafter, specimens were subjected to water spray for 15 sec followed by ultrasonication for 5 min to stop any further demineralization. After that, enamel specimens (*n* = 3) were subjected to microcomputed tomography (micro-CT) to verify the lesion formation before initiating the subsequent experimental processes.

Enamel specimens were randomly assigned to five different treatments groups: (1) HIFU exposure without CPICs nanoparticles application [HIFU/-NP], (2) CPICs nanoparticles application without HIFU exposure [NP/-HIFU], (3) CPICs application followed by HIFU exposure for 2.5 minutes [NP/ HIFU (2.5 min)], (4) CPICs application followed by HIFU exposure for 5 minutes [NP/ HIFU (5 min)], (5) and CPICs application followed by HIFU exposure for 10 minutes [NP/ HIFU (10 min)]. Enamel specimens having WSL without any intervention and sound enamel (WSL free) specimens were used as controls.

Briefly, the CPICs-ethanol solution (1ml; 2mg/ml) was applied to fully cover the WSL area and air-dried for 15 min at room temperature. Each enamel specimen was fixed the the bottom of a petri-dish filled with distilled water and then subjected to HIFU (Appendix Fig. 1) at the pre-determined exposure times (2.5 min, 5 min, or 10 min). For Group (2; [NP/-HIFU]), the WSL treated with CPICs were immersed in distilled water (without HIFU exposure) and incubated at 37 °C for 10 min (corresponding to the maximum duration of HIFU exposure used). The exposure time points (2.5 min, 5 min, or 10 min) for the enamel remineralization experiment were selected to be within a clinically reasonable intervention timeframe and also based on the results of the previous experimental stage investigating the effect of HIFU on HAP formation from CPICs placed on an amorphous glass substrate (*Effect of HIFU on HAP formation).*

***Enamel ultrastructural and crystallographic changes***

The surface morphologies of the experimental and control enamel specimens were viewed using a scanning electron microscope (SEM; Verios XHR SEM, Thermo Fisher Scientific, US) (*n=*5/group). Cross-sectional SEM images were obtained by fracturing each enamel specimen through the treatment window as previously described (Hua et al. 2020). The specimens were mounted on aluminium stubs with copper tape and sputter coated with platinum. Secondary electron images were acquired at different magnifications using an Everhart-Thornley detector at an accelerating voltage of 10 kV.

The surface topography and roughness of the treated enamel specimens were further investigated by atomic force microscopy (AFM; Cypher VRS, Oxford Instruments, UK). AFM was performed in tapping mode using a platinum-coated beam-shaped cantilever (HQ: XSC11/Al BS; MikroMash; Bulgaria). AFM images were obtained with a field view of 25 µm × 25 µm and 256 × 256 pixels per image (*n* = 5/per group). The surface roughness parameter (S_a_) was calculated using Gwyddion software (Gwyddion 2.62; Czech Metrology Institute, CZ). The measurements were repeated for seven different areas of 25 µm X 25 µm.

To identify the mineral phases and composition, enamel specimens, from each group (n = 3), were characterized by X-ray diffraction (XRD). The XRD diffraction peaks of the enamel specimens having WSL were first recorded over a 2θ range of 10 – 60° with an increment of 0.0110° using Aeris benchtop X-ray diffractometer (Malvern Panalytical, UK) with cobalt radiation (λ= 1.78 A°) at 40 kV and 15 mA. Enamel specimens treated with CPICs before and after the subsequent HIFU exposure were recoded to investigate any crystallographic changes induced by the remineralization process.

***Minerals composition/density and nano-mechanical properties***

The enamel specimens from the experimental and control groups (*n* = 5/group) were characterized by Raman microscope (WITec alpha 300RA; Germany) equipped with a laser with an excitation wavelength of 785 nm for studying the minerals composition and content. Firstly, the instrument was calibrated using a silicon wafer to produce a standard Raman peak at 520 cm ^1^. Enamel specimens, from each group, were placed on glass slides and spectra were obtained at 20 × magnification. The spectrum of each enamel specimen was collected with an integration time of 1 sec and summing twenty repeat acquisitions from each location. After acquisition, all spectra were analysed using Project Five software (version 5.1, WITec). Each acquisition was repeated at ten different locations, and the average peak intensity across the ten measurements was reported. The Lorentz fitting function was performed to extract the area under curves of the band of the peak at 1070 cm^-1^ and 960 cm^-1^ utilizing Project Five software. Furthermore, Raman mapping utilizing the same equipment was performed to examine the distribution of minerals at 960 cm ^-1^. The peak at this wavelength was chosen because it is the strongest signal in the Raman spectrum of enamel and is considered as an indicator of the mineral content (Mohanty et al. 2013). The mapping was performed in three different locations at 25µm × 25µm and integration time for 0.2 sec of each specimen. After selecting the peak of interest, the Sum filter was used to create the image that represents the sum of intensity within the highlighted peak of interest.

To assess the mineral density and lesion depth, the experimental and control groups were scanned using micro-CT (Nikon’s XT H 225 ST CT, Japan) (*n*=6/group). The Helical micro-CT scanning techniques were used with the following imaging setting: 85 kV (voltage), 51 µA (current), and 4.3 W (power), 5.2 µm pixel size (resolution), 34.442 geometric magnification, 2.83 sec exposure time per 0.6° projection step, 24.0 dB (gain), 360 ° rotation, 1200 number of projections and without filters. Each time the specimens were positioned in the same way before and after the respective treatment intervention. The images were then reconstructed and beam hardening correction (preset 3) was done using CTPro3D software (Nikon's CTPro3D Version XT 6.7, Japan). To calculate mineral density, firstly, micro-CT Hounsfield unit (HU) phantom was calibrated utilising same tube (filled with water; water phantom) and the image setting parameters utilized for scanning and reconstruction of enamel specimens. The HU values for air and water were assigned as -1000 and 0, respectively to calibrate the Hounsfield unit scale in CTAn software (Version 1.14.4.1, Bruker micro-CT, Belgium), following the company protocol. Thereafter, mineral density (MD) was estimated from the projection data set using CTAn software, where the regions of interests (ROIs) were selected from three different sections of the WSL. The lesion depth was further measured from the same ROIs slice. For each section, twenty-five slices were selected to create volume of interest (VOI), where the average MD in HU was measured. In addition, the variation in lesion depth before and after treatment was measured from the same ROI slices. Furthermore, the remineralization percentage of the experimental groups were calculated using a previously described formula (Abdalla et al. 2023):

% Remineralization = (ΔZd – ΔZr/ΔZd) × 100%

Where ΔZd: the difference in MD between sound enamel and demineralized enamel, and ΔZr: the difference in MD between remineralized enamel and sound enamel***.***

The nanoindentation properties of the enamel specimens (*n=5* per group) were characterized using A DualScope^TM^ Scanning Probe & Optical Microscope (DME, Denmark), equipped with a Berkovich diamond indenter (tip radius ~20 nm). An array of 7 indents/lines separated by a 10 µm interval in between on an enamel specimen at five different locations was employed before and after remineralization. The enamel specimens were indented to a load force of 0.5 mN, holding time of 10 s between 20 increments and 20 decrements. Subsequently, the nano-hardness (H) and reduced-elastic modulus were calculated using the load function profile average at a compliance of 0.000360 µm/mN.

**HIFU effect on cariogenic biofilm**

***Cultivation of streptococcus mutans on enamel specimens***

*Streptococcus mutans* ***(****S. mutans*; ATCC 700610) was used for evaluating the anti-biofilm effect of HIFU on enamel specimens. The bacterial strains were cultured in brain-heart infusion (BHI) medium at 37 °C with 5% CO_2_ for 24 h and adjusted to 0.5 McFarland standard (1×10^8^ CFU/ml). The prepared enamel specimens were autoclaved at 121 °C for 15 min and placed into sterile 48-well plates followed by inoculating with 100 µl of *S. mutans* and 900 µl of BHI broth supplemented with 1% sucrose, followed by incubating anaerobically at 5% CO_2_ at 37 °C for 48 h to obtain a dense biofilm. After incubation, the specimens were rinsed thrice with PBS to remove non-adherent bacterial cells and was exposed to HIFU for the pre-determined time-points (2.5 min, 5 min, and 10 min). *S. mutans* biofilm formed on enamel specimens having WSL without HIFU exposure were used as control.

***Confocal Laser scanning microscopy (CLSM)***

The control and the experimental groups were stained for 15 min using LIVE/DEAD BacLight Viability Kit (ThermoFisher Scientific, USA) as per the manufacturer’s recommended instructions. Styo9 stains live bacterial cells and emits green fluorescence, while propidium iodide stains dead bacterial cells and emits red fluorescence. The stained specimens (n = 3/group) were rinsed with PBS, fixed with 4% paraformaldehyde for 10 min, and observed by CLSM (Nikon A1 Si confocal microscope, Nikon Instruments Inc., USA).

***MTT assay***

The metabolic activity of the *S. mutans* biofilms was evaluated by the 3-(4,5-Dimethylthiazol-2-yl)-2,5-diphenyltetrazolium bromide (MTT) assay using an MTT bromide kit (0.5 mg/mL MTT solution; Sigma-Aldrich, Australia). The control and the experimental specimens (*n* = 5/group) were ultrasonicated, then 200 µL of the bacterial suspension was transferred into a 96-well plate in triplicates, followed by adding 20 µL of the MTT reagent into each well and incubated at 37 °C for 4 h. The reagent was then aspirated and replenished with 200 µL of the solubilizing solution and incubated overnight at 37 °C. The absorbance of the supernatant was measured with a spectrophotometer (Sunrise, Tecan, Switzerland) at 570 nm.

***Colony forming unit assay***

For further confirmation, a colony forming unit (CFU) assay was performed as described previously with slight modification (Rajan et al. 2023). The enamel specimens (*n* = 5/group) were placed in a vial containing 1 ml of sterile distilled water followed by ultrasonication to release attached bacteria. Then, the obtained solution was serially diluted, and 100 µl of the suspension was plated on the BHI agar plates and incubated at 37 °C overnight. Then the number of viable colonies was counted.

**Statistical analysis**

The normal distribution of data was investigated by Shapiro–Wilk test. All data are presented as the mean ± standard deviation. One way -ANOVA followed by post-hoc Tukey’s test was performed to determine the statistical differences among the groups at the alpha level (*p* < 0.05). All statistical tests were performed using statistical package for the social sciences (SPSS; Version 23.0, IBM Armonk, NY, US).

**Results**

***Antibiofilm Effect of HIFU***

The confocal laser microscopy (CLSM) showed the formation of a well-established biofilm layer comprising living bacteria, fully covering the enamel specimens having WSL (Appendix Fig. 1B). However, following HIFU exposure, the bacterial biofilms started to be eradicated from the enamel surface, and this removal effect was time-dependent (Appendix Fig. 1 C-E). No viable bacterial cells can be seen on the enamel surface following HIFU exposure for 5 and 10 min (Appendix Fig. 1D, E). This observation was corroborated by the colony forming unit (CFU) assay, demonstrating a significant reduction in CFU count with increasing HIFU exposure time (Appendix Fig. 1 F-J). Additionally, a significant decrease in the metabolic activity of *S. mutans* biofilm was observed with prolonged HIFU exposure, indicative of impaired mitochondrial function (Appendix Fig. 1 K).


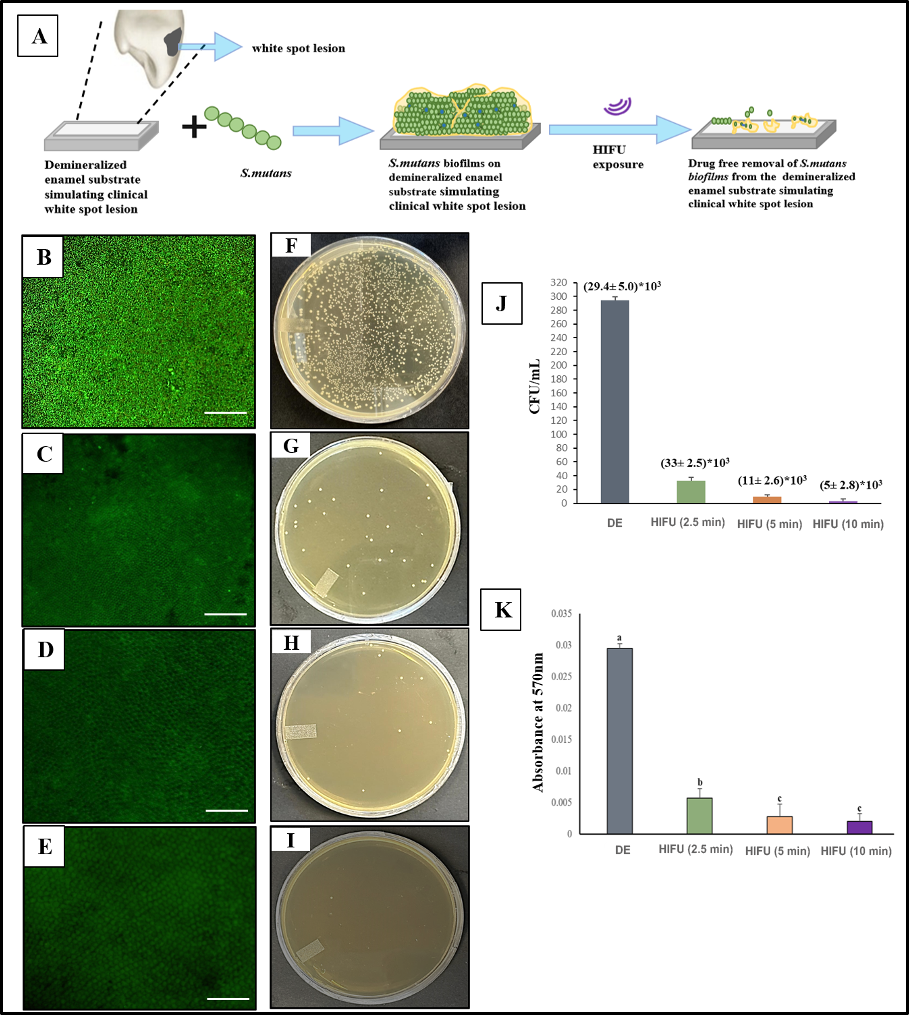


**Appendix Figure 1.** (A) Schematic illustration of the proposed principal drug-free anti-biofilm effect of HIFU against cariogenic bacteria *(S. mutans)* attached to enamel specimens having WSL. CLSM images of (B) WSL specimens fully covered by *S. mutans* biofilms, with green staining indicating live bacteria. (C-E) CLSM images of enamel specimens with WSL following HIFU exposure for 2.5 min (C), 5 min (D), and 10 min (E), demonstrating the eradication of *S. mutans* biofilm which is dependent on HIFU exposure time. (F) CFU plates displaying the dense viable bacterial colony formed on the surface of the control enamel specimens (WSL). (G-I) However, a clear reduction in bacterial colonies can be seen in CFU plates following HIFU exposure for (G) 2.5 min, (H) 5 min, and (I) 10 min. This reduction was significantly dependent on HIFU exposure time (J). (K) Bar chart showing the significant reduction in the relative metabolic activity of *S. mutans* biofilms with the increase in HIFU exposure time. Dissimilar letter indicates statistically significant difference (*p* < 0.05). Scale bars represent 50 µm.

**References**

Aati S, Akram Z, Shrestha B, Patel J, Shih B, Shearston K, Ngo H, Fawzy A. 2022a. Effect of post-curing light exposure time on the physico–mechanical properties and cytotoxicity of 3d-printed denture base material. Dent Mater. 38(1):57-67.

Aati S, Shrestha B, Fawzy A. 2022b. Cytotoxicity and antimicrobial efficiency of zro2 nanoparticles reinforced 3d printed resins. Dent Mater. 38(8):1432-1442.

Abdalla MM, Bijle MN, Abdallah NM, Yiu CKY. 2023. Enamel remineralization potential and antimicrobial effect of a fluoride varnish containing calcium strontium silicate. J Dent. 138:104731.

He J, Yang J, Li M, Li Y, Pang Y, Deng J, Zhang X, Liu W. 2022. Polyzwitterion manipulates remineralization and antibiofilm functions against dental demineralization. ACS nano. 16(2):3119-3134.

Hua F, Yan J, Zhao S, Yang H, He H. 2020. In vitro remineralization of enamel white spot lesions with a carrier-based amorphous calcium phosphate delivery system. Clin Oral Investig. 24:2079-2089.

Mohanty B, Dadlani D, Mahoney D, Mann A. 2013. Characterizing and identifying incipient carious lesions in dental enamel using micro-raman spectroscopy. Caries Res. 47(1):27-33.

Rajan SM, Shrestha B, Aati S, Kujan O, Tay A, Fawzy AS. 2023. Evaluation of antibacterial efficacy of high-intensity focused ultrasound versus photodynamic therapy against enterococcus faecalis–infected root canals. Ultrasound Med Biol.

Shao C, Jin B, Mu Z, Lu H, Zhao Y, Wu Z, Yan L, Zhang Z, Zhou Y, Pan H. 2019. Repair of tooth enamel by a biomimetic mineralization frontier ensuring epitaxial growth. Sci Adv. 5(8): eaaw9569.
